# Quality specifications in postgraduate medical e-learning: an integrative literature review leading to a postgraduate medical e-learning model

**DOI:** 10.1186/s12909-016-0700-7

**Published:** 2016-07-08

**Authors:** R. A. De Leeuw, M. Westerman, E. Nelson, J. C. F. Ket, F. Scheele

**Affiliations:** Athena Institute for Trans-disciplinary Research, VU University Amsterdam, De Boelelaan 1118, Amsterdam, 1081 HZ The Netherlands; VUmc, School of Medical Sciences, Amsterdam, The Netherlands; Division of Pediatric Infectious Diseases, Department of Pediatrics, Stanford University School of Medicine, Stanford, USA; VU University Amsterdam, University Library, Amsterdam, The Netherlands

**Keywords:** E-learning, Distance learning, Education, Medical education, Medical e-learning, Quality model, Integrative review, Postgraduate medical education

## Abstract

**Background:**

E-learning is driving major shifts in medical education. Prioritizing learning theories and quality models improves the success of e-learning programs. Although many e-learning quality standards are available, few are focused on postgraduate medical education.

**Methods:**

We conducted an integrative review of the current postgraduate medical e-learning literature to identify quality specifications. The literature was thematically organized into a working model.

**Results:**

Unique quality specifications (*n* = 72) were consolidated and re-organized into a six-domain model that we called the Postgraduate Medical E-learning Model (Postgraduate ME Model)*.* This model was partially based on the ISO-19796 standard, and drew on cognitive load multimedia principles. The domains of the model are preparation, software design and system specifications, communication, content, assessment, and maintenance.

**Conclusion:**

This review clarified the current state of postgraduate medical e-learning standards and specifications. It also synthesized these specifications into a single working model. To validate our findings, the next-steps include testing the Postgraduate ME Model in controlled e-learning settings.

## Background

E-learning plays a prominent role in conventional education, adult education, and medical training because of its flexibility, broad resource-sharing capacity, and cost-effective scalability [1]. E-learning has become central to medical education, and web technologies offer valuable new opportunities for both under- and postgraduate medical education [2]. E-learning also offers participants’ an advantage in that they can choose a comfortable and accessible place and time to study, which is important in postgraduate and continuous medical education [3].

There are many studies comparing e-learning methods. However, one of the problems is that the results of studies directly comparing technology-assisted education with traditional teaching often conflict and often do not demonstrate or propose best practices [4, 5]. Critical evaluation of the quality and efficiency of e-learning is warranted [6]. Therefore, there is a need to develop a consensus-based quality assurance standard for postgraduate medical e-learning [7, 8].

It is known that e-learning should be targeted to the needs of the specific audience (in this case postgraduates) [9, 10]. The success of e-learning programs has also been linked to the use of a theoretical framework or a learning theory [11]. Standards for e-learning exist and have been evaluated [12].

Although learning theories are broad and diverse, there is progressive agreement about the psychological fundamentals [13]. Previous research suggests that the constructivist approach (founded by Jean Piaget, among others) is compatible and appropriately designed for e-learning. According to this theory, humans are active learners and construct new knowledge based on prior experiences and interactions [14]. An example is problem-based learning, which has been shown to be effective in medical education [15, 16]. Another theory based on the constructivist approach is the cognitive load theory (CLT) developed by Sweller [17]. The constructivist approach also forms the foundation of the cognitive theory of multimedia learning, a well-evaluated learning theory developed by Mayer [18]. This theory has been specifically adjusted for e-learning, and is believed to provide a good basis for an e-learning standard [18]. These theories are not elaborated here. However, it is important to remember that learning theory, not technology, should guide the design and content of e-learning.

Standards for e-learning exist [12], but are often isolated to a specific sector of the industry for which they were developed [19]. There are many industrial standards in education, often published by organizations such as the American Council on Education and the European Association of Distance Teaching Universities [20]. Several organizations have made progress in developing international industrial standards for universities, including the Open e-Quality Learning Standards and the Leonardo DaVinci program [21, 22].

The most common formal standard in use is the ISO/IEC 19796-1 [23]. This standard was issued by the International Standardization Organization (ISO) in 2005, and contains the Reference Framework for the Description of Quality Approaches (RFDQ), a framework supported by the European Quality Observatory [24]. Stracke implemented this standard and concluded that it was not only the first quality standard for learning, education, and training, but was a valuable instrument for sustained quality development in e-learning [23]. Little tested two higher education quality standards or rubrics, in professional continuous nursing education in 2009 [25]. The College of Public Health Online Course Standards and the Quality Matters Peer Course Review Rubric were evaluated, and although they look hopeful, little is known about their actual effectiveness. The Quality Matters rubric is not publicly available, making evaluation even less accessible.

Existing literature fails to clarify the methods and strategies used to evaluate the quality of postgraduate medical e-learning. As Clark noted in his well-respected book *E-Learning and the Science of Instruction*, the target audience should be the first thing considered when designing an e-learning program [9, 10]. Although undergraduates and postgraduates may learn in the same way, adult learning theories suggest their learning strategies and capacities might be dissimilar. Therefore, we focused our review on our target audience*:* postgraduates and physicians bound to continuous medical education. Ellaway et al. began this process in 2008 by describing a two-part guideline for e-learning in medical education. Although the guideline was not specifically aimed at postgraduate medical education, it served as a foundation for further research [16, 26]. The previously mentioned standards and the Quality Matters Rubric look promising, but are all aimed at different target audiences [25]. The ISO standard (ISO/IEC 19796) covers a lot of domains, but lacks detail regarding their application [27].

It is difficult to establish quality control practices for e-learning materials [28], yet this is an important problem in postgraduate medical education where the quality of training directly influences patient care [29]. Therefore, as technological innovations reshape medical education institutions, the question of quality assurance is at the forefront of university leadership concerns worldwide [30]. In 2010, Cook et al. reiterated the primary importance of defining quality standards in medical education. In their Second Flexner Report, they identified standardization as a key goal for improving medical education [31]. We believe that it is important to establish a testable quality assurance model to improve the uptake of e-learning and motivate continuous medical learning [5]. This review will add to existing literature by providing an integrative literature review and a working model of quality assurance standards in postgraduate medical e-learning.

## Methods

We performed an integrative review to identify and critically evaluate qualitative and quantitative literature associated with current postgraduate medical e-learning quality assurance. We used the updated integrative review methodology developed by Whittemore et al. [32]. This method consists of three steps: 1) a systematic search combined with at least one nonsystematic search method; 2) data evaluation comparing study models and quality scores; and 3) data analysis. During data analysis, we compared individual quality items, clustered them, searched for contrasts and comparisons, discerned patterns, and built an overview of the domains.

### Definitions

#### E-learning

An approach to teaching and learning, representing all or part of the educational model applied, based on the use of electronic media and devices as tools for improving access to training, communication, and interaction and that facilitates the adoption of new ways of understanding and developing learning [33]. In practice, the definition includes any digital content made to teach and distributed physically or online.

#### Quality

To date, there is no consensus definition of quality. However, a high-quality product is generally defined as one that meets consumer-defined specifications, delights the consumer, consistently meets the standard that the producer has set for itself, and leads to customer satisfaction. Producers should be able to assure this quality [30, 33].

#### Postgraduate (and continuous) medical education

Any form of learning aimed at medical professionals who have graduated from formal training and residency, or used by medical professionals a part of continuous learning to maintain their competency and develop new knowledge [34].

#### Standards and specifications

A *standard* is a set of specifications that guide an e-learning author in developing an e-learning program. A *specification* is a specific item that is addressed within the standard [17].

### Step 1: Systematic literature search

The primary search terms were *distance learning* (and all synonyms) [35] and *quality* (and all synonyms). We conducted the search on May 2, 2015. We searched ISI/Web of Knowledge, PubMed, EBSCO/Cinahl, EBSCO/PsycInfo, and EBSCo/ERIC (Table 1). Google Scholar was also searched, despite debate on its added value [36].

**Table 1:** Databases searched and corresponding search strings

Database	Search string used
PubMed	(((((assessment[Title/Abstract] OR criteria[Title/Abstract] OR metrics[Title/Abstract] OR characteristics[Title/Abstract] OR measurement[Title/Abstract] OR evaluation[Title/Abstract] OR “Quality Control”[MeSH] OR “Quality Improvement”[MeSH] OR “standards”[Subheading] OR “Guideline”[Publication Type] OR “Guideline Adherence”[MeSH])) OR quality[Title/Abstract]) OR principles[Title/Abstract])) AND ((“Education, Distance”[MeSH] OR e-learning[Title/Abstract] OR electronic learning[Title/Abstract] OR distance education[Title/Abstract] OR technology-enhanced learning[Title/Abstract] OR tele-learning[Title/Abstract] OR web-based learning[Title/Abstract] OR web-based education[Title/Abstract] OR internet-based learning[Title/Abstract] OR computer based learning[Title/Abstract] OR computer-assisted instruction[Title/Abstract] OR distance learning[Title/Abstract] OR online learning[Title/Abstract] OR ((“Learning”[MeSH] OR “Education, Professional”[MeSH]) AND (“Computer Communication Networks”[MeSH] OR “Computer-Assisted Instruction”[MeSH]))))
ISI/Web of Knowledge	(assessment OR criteria OR metrics OR characteristics OR measurement OR evaluation OR standards OR quality OR principles) [TI] AND (e-learning OR (electronic learning) OR (distance education) OR (technology-enhanced learning) OR tele-learning OR (web-based learning) OR (web-based education) OR (internet-based learning) OR (computer based learning) OR (computer-assisted instruction) OR (distance learning) OR (online learning)) [AB]
EBSCO/Cinahl	(assessment OR criteria OR metrics OR characteristics OR measurement OR evaluation OR standards OR quality OR principles) [AB] AND (e-learning OR (electronic learning) OR (distance education) OR (technology-enhanced learning) OR tele-learning OR (web-based learning) OR (web-based education) OR (internet-based learning) OR (computer based learning) OR (computer-assisted instruction) OR (distance learning) OR (online learning)) [AB]
EBSCO/Psychinfo	(assessment OR criteria OR metrics OR characteristics OR measurement OR evaluation OR standards OR quality OR principles) [AB] AND (e-learning OR (electronic learning) OR (distance education) OR (technology-enhanced learning) OR tele-learning OR (web-based learning) OR (web-based education) OR (internet-based learning) OR (computer based learning) OR (computer-assisted instruction) OR (distance learning) OR (online learning)) [AB]
EBSCO/Eric	(assessment OR criteria OR metrics OR characteristics OR measurement OR evaluation OR standards OR quality OR principles) [AB] AND (e-learning OR (electronic learning) OR (distance education) OR (technology-enhanced learning) OR tele-learning OR (web-based learning) OR (web-based education) OR (internet-based learning) OR (computer based learning) OR (computer-assisted instruction) OR (distance learning) OR (online learning)) [AB]
Google Scholar	All in title: (assessment OR criteria OR metrics OR characteristics OR measurement OR evaluation OR standards OR quality OR principles) AND ((e-learning OR (electronic AND learning) OR (distance AND education) OR (technology-enhanced AND learning) OR tele-learning OR (web-based AND learning) OR (web-based AND education) OR (internet-based AND learning) OR (computer AND based AND learning) OR (computer-assisted AND instruction) OR (distance AND learning) OR (online AND learning))

#### Inclusion criteria

Included articles were peer-reviewed and published in the English language between 1970 and May 2015. The articles had to describe and evaluate specific e-learning characteristics in postgraduate or continuous medical education. The search was kept broad and selection was made on postgraduate medical education manually after reading the titles, abstracts, and the full text (if necessary).

#### Exclusion criteria

We excluded dissertations, conference abstracts, and articles comparing e-learning with other forms of learning without describing the quality specifications used.

After selecting the titles and abstracts, we sampled 40 random abstracts for independent evaluation by a second (MW) and third author (FS). If there was no consensus, three authors participated (RL, MW, FS) in a discussion until consensus was reached on whether or not to include the article.

### Step 2: Data evaluation

We aggregated the selected studies that met the inclusion criteria, flagging those with an unclear method. The aggregate was graded according to criteria adapted from the levels of evidence model of the Oxford Centre for Evidence-Based Medicine (Table 2). Our adaptation consisted of the addition of grade 6, indicating an unclear method. We flagged a study as unclear if it did not fall into any of the five existing categories.

**Table 2:** Adapted Oxford Centre for Evidence-Based Medicine levels of evidence

Level	Design	Tier
1	Systematic review of randomized controlled trials or individual randomized controlled trial	high
2	Systematic review of cohort studies or individual cohort study	high
3	Systematic review of case-control studies or individual case-control study	high
4	Case-series	low
5	Expert opinion	low
6	Unclear method	low

We used the two-tier data evaluation strategy developed by Wittemore et al*.* to grade the quality of each item. Levels 1, 2, and 3 were identified as high quality (tier 1), and 4 and 5 as low quality (tier 2) [32]. We removed grade 6 studies from the analysis.

### Step 3: Data analysis

After aggregating the studies and grading the papers, we analysed each item for common themes and contradictory findings. If items conflicted, we rejected the lower quality study in favor of the higher quality study. We categorized the items according to the ISO/IEC 19796-1 domains, and then generated new themes for items that did not fit the established domains.

## Results

### Primary selection and analysis

The literature search identified 10,732 articles. Searching Google Scholar gave us access to unanticipated databases such as the Emerald, IEXEE, and Editlib, as well as journals that were not registered with the other databases (International Journal of Information and Educational Technology, Journal of E-Learning and Knowledge Society, Applied Soft Computing, International Educational E-Journal, and Journal of Theoretical and Applied Information Technology). We then manually reviewed the identified studies.

After applying inclusion and exclusion criteria, 884 eligible titles remained. Eligible abstracts were reviewed and final selection criteria were refined as described in the Methods section.

**Fig. 1:**
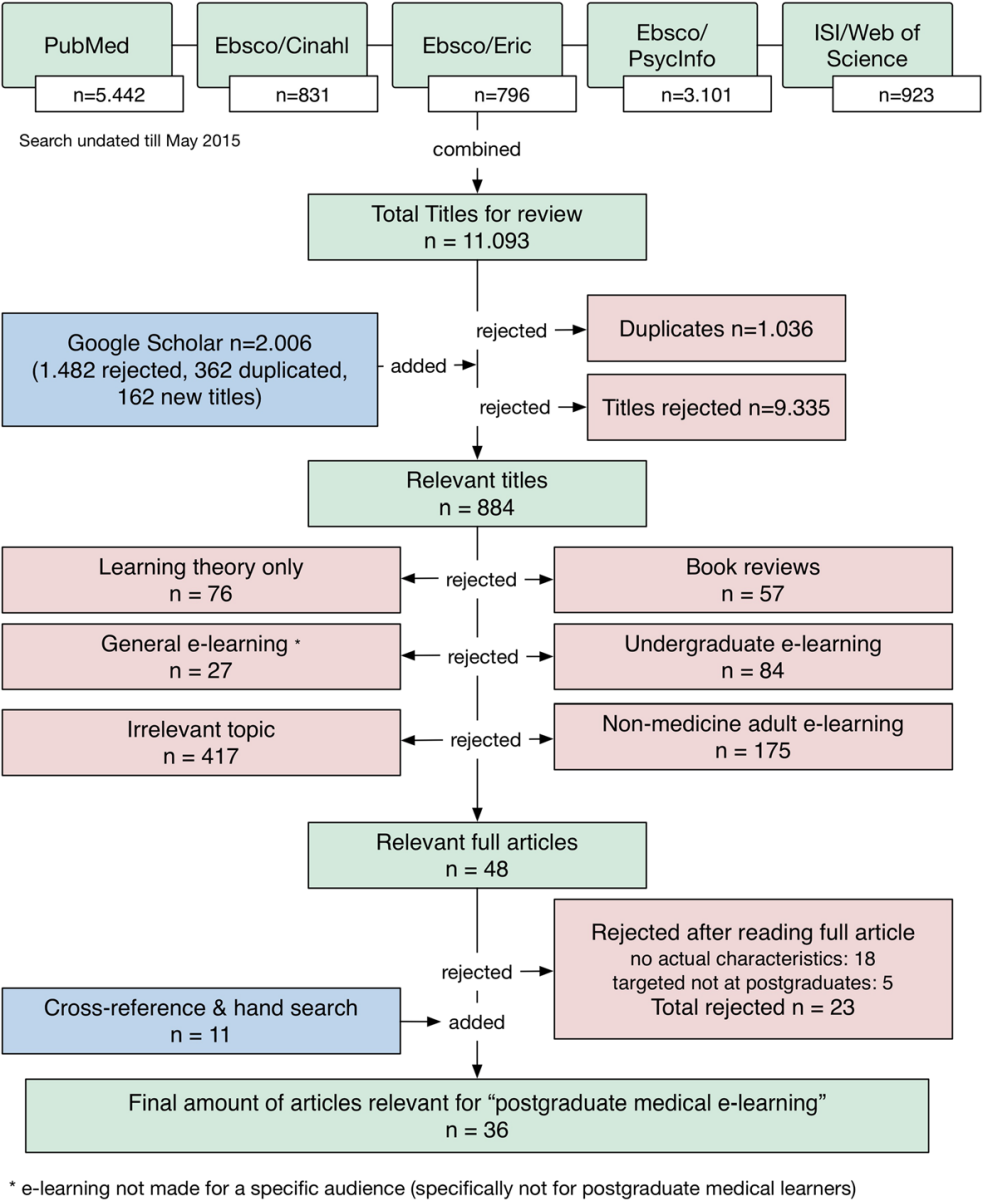
Flowchart of the literature search results

In total, 36 articles met the final selection criteria (see Fig. 1), representing quality items in postgraduate medical education literature. Of these, 15 were original case reports and expert opinions; 13 were reviews of previous case reports and expert opinions; five were randomized controlled trials; and two were meta-analyses (Table 3). Collectively, these articles represented 16 high tier articles (Oxford Centre for Evidence-Based Medicine levels 1, 2, and 3) and 20 low tier articles (levels 4 and 5). The publication dates ranged from 1995 to 2015.

**Table 3:** Articles used for the postgraduate medical education model identified in the literature search

First author	Year	Type of study	Domains discussed*	Evidence tier	Reference
Khoiny	1995	Expert opinion	1	Low	[57]
Kim	1999	Systematic review	1, 2, 4, 5, 6	High	[72]
Sekikawa	2001	Expert opinion	2	Low	[73]
Doyle	2002	Expert opinion	2, 3	Low	[74]
Jha	2002	Case study	1, 2, 4	Low	[75]
Minasian	2002	Expert opinion	1, 2, 5, 6	Low	[55]
Cook	2004	Review	1, 2, 3, 4, 5, 6	high	[6]
Olson	2004	Review	1, 2, 3, 4, 5, 6	high	[20]
Knight	2004	Expert opinion	1, 2, 3, 4, 5, 6	low	[48]
Casebeer	2004	Trial	2, 4, 5	high	[44]
Curry	2005	Expert opinion	1, 2, 4, 5, 6	low	[47]
Bangert	2005	Case study	2, 3, 4, 5	low	[46]
Garde	2007	Case study	3, 4, 5	low	[43]
Maor	2007	Review	3, 4	high	[45]
Posel	2009	Review	1, 2, 4, 5, 6	high	[66]
Casimiro	2009	Review	2, 4, 5	high	[71]
Cook	2010	RCT	3, 4, 5	high	[50]
Wong	2010	Review	1, 2, 3, 4, 5	high	[51]
Mayer	2010	Review	4	high	[54]
Short	2010	Review	2, 3, 4, 5	high	[64]
Alexander	2010	Expert opinion	1, 2, 3, 5, 6	low	[37]
Friedlander	2011	Review	4	high	[62]
Chang	2011	Case study	1, 2, 3, 4	low	[49]
Issa	2011	Cohort study	4	low	[63]
Mounsey	2012	RCT	4	high	[60]
Raymond	2012	Expert opinion	1, 3, 4, 5	low	[41]
Sowan	2013	Expert opinion	1, 3, 4, 5	low	[39]
Mhouti	2013	Review	1, 3, 4, 5	high	[58]
Bluestone	2013	Meta-analysis	4, 5	high	[61]
Gordon	2013	RCT	4	high	[38]
Shaw	2014	Case study	2, 4, 5	low	[76]
Lewis	2014	Review	1, 2, 4, 5	high	[52]
Yavner	2014	Expert opinion	1, 2, 4, 5, 6	low	[53]
Lau	2014	Review	2, 4, 5	high	[42]
Davids	2014	RCT	2, 4, 5	high	[56]
Cook	2015	Review	1, 2, 3, 4, 5, 6	high	[10]

*RCT* randomized controlled trial

Domains: 1. Preparation, 2. Design and system, 3. Communication, 4. Content, 5. Assessment, 6. Maintenance

### Sub-selection and analysis

**Fig. 2:**
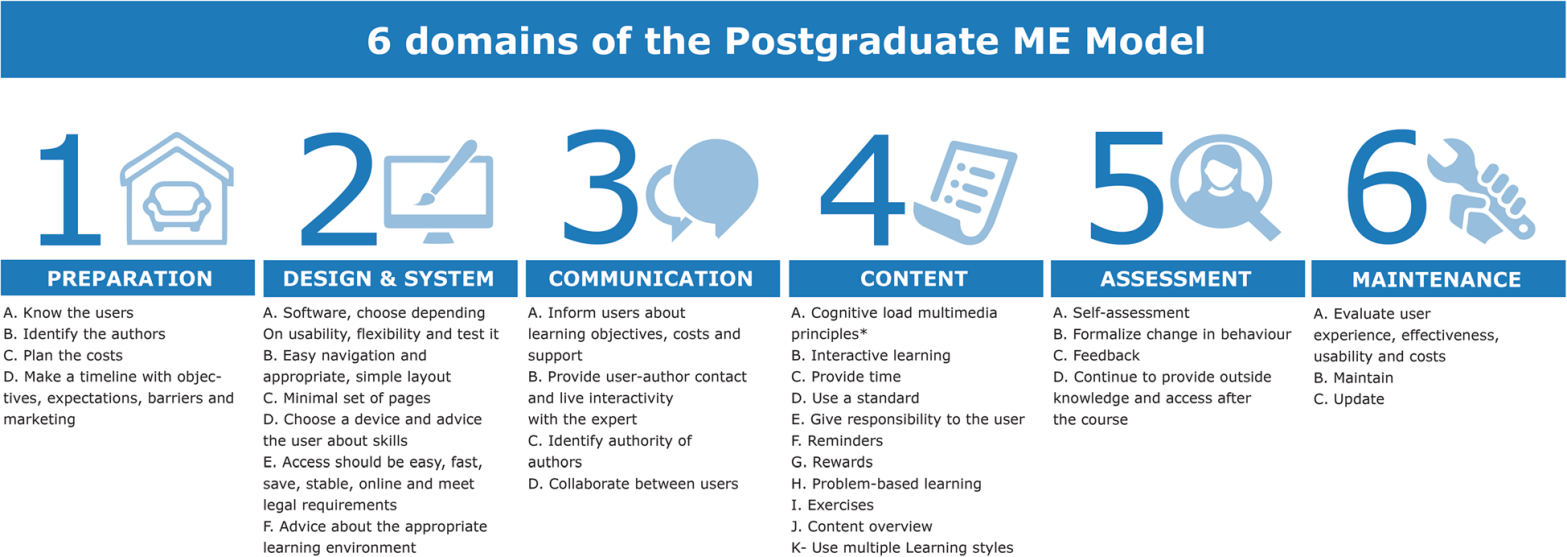
Postgraduate medical e-learning specifications model

After consolidating duplicates for quality specifications (440 in total), there were 72 unique quality specifications remaining. We categorized these specifications according to the seven ISO 19796 domains, combined them, and renamed them if necessary. This produced a model with six final domains, which we called the Postgraduate Medical E-learning Model (Postgraduate ME Model; Fig. 2). In the following paragraphs, we have defined the domains and provided examples from the literature.

1. **Preparation:** This step should be performed before designing and building the e-learning platform. Twelve articles described the importance of knowing the end users [23, 36–46]. Preparation is a two-step process. First, one must define the platforms used by different types of end users and their expectations of the e-learning platform. If the end users’ level of knowledge is known, the e-learning strategy can be designed appropriately. Special functionalities for individuals with learning disabilities can also be incorporated if applicable. Cook et al. described this step as a part of the needs analysis [10]. If possible, learning should be adapted to the audience’s motivational level, skills, and expectations. Curry et al*.* [47] and Olson et al*.* [20] emphasized the importance of selecting e-learning authors based on subject matter expertise. They suggested that academically qualified people must actively participate in development and training to improve e-learning. Four articles advised readers to allocate time to accurately budget and plan for the expected costs [23, 41, 44, 48]. Budgets and policy statements should reflect commitment to the program. Seven authors agreed on the positive effect of planning, describing objectives and expectations, and placing these in a timeline to maximize efficiency [7, 23, 36, 37, 39, 40, 49]. Cook et al. [50] identified potential barriers to implementation, and Olson et al. [20] advised readers to develop a marketing plan for reaching end users. Curry [47], Wong [51], and Sowan [39] advocated efficient e-learning that saves the user time, compared with other forms of learning.
2. **Design:** This depends on a series of difficult choices that include the types of hardware and software that best fit end-users’ needs*.* Although, hardware and software are very different, most articles described these in one section. In several articles (*n* = 5), authors stressed the importance of reliability and emphasized that the combined hardware and software system should be tested and iterated to meet end-user design requirements [7, 23, 39, 42, 49]. Testing should include different browsers and different monitors with varying resolutions, as well as different hand-held devices if a mobile-based platform is developed.
  Most (*n* = 14) articles discussing software design focused on ready access to the e-learning platform. Access should be fast, easy to find, and always available; the platform should have a short loading time, and should provide reliable online access to all external links [7, 36-40, 45, 46, 48, 49, 52, 53, 70]. A secure connection is also important to support the privacy and legal requirements, copyright rules, and intellectual property issues [23, 39, 44, 49]. Lewis et al. [52] urged readers to be mindful of the basics such as grammar and spelling. Cook et al. [6] advised piloting e-learning websites before publishing them, and Bangert [46] advised the use of a variety of learning environments. Yavner et al. [53] wanted to give users maximum control over navigation, an approach that Mayer later challenged [54]. Navigation and layout are important design elements. Navigation should be user friendly and intuitive with a “less is more” design strategy. The design should be visually pleasing, adhere to the principles of excellent website design, and use reusable learning objects for a standard look and feel [37, 45]. Four articles provided a minimal set of pages/functionalities: glossary of terms, frequently asked questions, concept map, references cited, abbreviation key, and labelled diagrams [36, 45, 55, 56].
  Olson et al. [20] and Lau [42] suggested choosing appropriate, intuitive, and user-friendly hardware devices that advise the learner of the skills and technology required for success. Desired learning outcomes should drive the choice of technology. Khoiny [57], Garde et al. [43], and Mhouti et al. [58] described the importance of the environment where the e-learning is used. The physical setting should be a stimulating and motivational place to learn.
3. **Communication:** This includes all forms of internal user-oriented communication and external expert-oriented communication. Articles included in our review provided descriptions of several forms of communication, including: (i) communication about the program that informs the user about learning objectives, costs, and support options; (ii) communication that allows users to contact the faculty/course authors; and (iii) communication between users as they collaborate on coursework. Several articles (*n* = 8) emphasized the second form of communication, recommending opportunities for live interaction with experts/authors, possibly in a group context [38, 41, 43, 45, 55, 59]. The credibility of the authors should be well established, and should include the authors’ credentials and disclosure of sponsors and conflicts of interest.
4. **Content:** This is the aggregate material used to build the lessons (e.g., words, images, videos). Content was a central theme in the articles we reviewed. Problem-based learning was favoured by 13 articles as the best way to incorporate motivation and better understanding [7, 15, 40, 43, 46–48, 52, 56–60]. Introducing interactivity to encourage higher-order thinking was also important (*n* = 16 articles) [23, 36, 39–41, 45–47, 51, 52, 56–59, 61]. The user should be provided with time and impetus to learn with as little stress as possible and made responsible for the learning process to create a feeling of belonging, and the platform should provide learning exercises [7, 41, 46, 48, 61]. Learning modules should end with summaries, consist of short paragraphs, state a timeline, and use milestones [23, 36, 39, 41, 43, 44, 46, 47, 49, 50, 59]. Lewis et al. [52] emphasized the use of educational standards, and most other articles suggested using cognitive load principles. Cognitive load principles are described in the Discussion. Bluestone et al. [61] advocated for reminders in e-learning systems, and Friedlander et al. [62] and Cook [59] suggested incorporating rewards and reinforcements to maintain motivation.
5. **Assessment:** This refers to all of the possible ways to test end users and formalize their knowledge gain. Almost all of the articles prioritized assessment and feedback on performance. Seventeen articles described assessment as most effective when used in a direct, continuous, and personalized way [7, 15, 23, 36–38, 40, 41, 45–47, 50–52, 56, 58, 63]. Self-assessment was also an important part of learning [7, 23, 36, 39, 40, 43–45, 47, 54, 57, 59]. Additionally, Lau [42], Short et al. [64], and Wong et al. [51] stressed the importance of continuing to provide the e-learning software and associated tools after the end of the course. Cook et al. focused on the importance of assessing user experience and satisfaction [50]. Assessment is also a way of evaluating learning outcomes [10].
6. **Maintenance:** This includes the steps taken to avoid the loss of knowledge after a user finishes the e-learning process. Maintenance also includes reliable long-term access to the platform to allow an end user to return to the platform. Maintenance-related articles (*n* = 10) described the importance of evaluating a platform’s user experience, effectiveness, usability, and cost [7, 23, 36–38, 40, 44–46, 59]. Technical maintenance included protecting and verifying hyperlinks. Several articles (*n* = 6) emphasized that modifications to e-learning programs should be enabled and updated, and those proven to be unsuccessful removed [7, 23, 37, 38, 44, 45]. E-learning developers should also estimate the reusability and sustainability of new platforms, as these factors are important in the platform’s maintainability [10].

## Discussion

We identified 72 features as important in postgraduate medical e-learning, and grouped these into six domains. The domains also provided a model framework for educators involved in drafting e-learning strategies or evaluating e-learning initiatives. The content domain is the most widely described and discussed domain of the model, and we discuss this in detail below followed by the limitations of our review.

### The content domain

Perhaps the most important part of e-learning is the content, which was emphasized in all articles reviewed. Content is the heart of e-learning, and the design merely delivers content. A common pitfall is developing e-learning simply for the sake of using a new technology. Instead of making e-learning technology-centred, developers should subscribe to a learning theory to ensure the design is guided by pedagogical principles [14]. Mayer described the cognitive theory of multimedia learning, based the CLT [17]. CLT aims to develop instructional design guidelines based on a model of human cognitive architecture [65]. CLT states that working memory is limited in its capacity to selectively attend to and process incoming sensory data. This theory is concerned with the way in which a learner focuses and uses cognitive resources during learning and problem solving. It suggests that for an instruction to be effective, it must be designed in a way that does not overload the mind’s capacity for processing information [40]. Based on this theory, Mayer defined a set of principles, which describe the effect of different design techniques on learning. These principles form guidelines for using multimedia in a learning environment. In the content domain, we found 20 of 36 articles tested or recommended one or more of Mayer’s principles. Because the current literature pays so much attention to these principles, we considered them to be the foundation of the content domain [18].

Not all authors agreed with all of these principles. For example, Yavner et al. [53] proposed giving the learner maximum control, in contradiction to Mayer’s assertion that giving control to learners yields no benefit because learners may have too many options [17]. Curry et al. [47], Mhouti et al. [58], and Posel et al. [66] also highlighted the importance of learner-centred e-learning and supported individualized, rather than standards-based e-learning. Therefore, consensus on the principles of the content of e-learning is lacking.

### Limitations

The major limitations of this review pertain to the methods. We performed an integrative review instead of a systematic review or meta-analysis, because the majority of published studies related to postgraduate medical education did not meet the parameters required for a systematic review. The major limitation of integrative reviews is the potential for bias from the inclusion of non-peer-reviewed information or lower-quality studies. Although Cook et al. [50] conducted a meta-analysis, it was limited to a few domains in e-learning. However, the literature from which the authors drew their conclusions was largely consensus-based.

The second limitation was the fast-changing technology that threatens to render our results obsolete. E-learning is rapidly changing the landscape of medical education and is developing faster than research can evaluate it [67]. This pace of change is a limitation of our review because research is always one step behind technology [67]. Examples of these fast changes are two disruptive innovations in medical education: Massive Open Online Courses and social media on mobile devices [68]. Both impact on e-learning and might dramatically change the education landscape [69]. In this landscape, it is almost impossible to evaluate an innovation properly before it is already outdated. In addition, social media is expected to become important to the collaboration domain of e-learning [70].

Despite these limitations, we believe that our six-domain Postgraduate ME Model will generate discussion and increase the quality of new e-learning courses. Our e-learning model could be interpreted as a general framework rather than postgraduate-specific, although we have not provided evidence to extend it to other settings, due to the limitation in our search strategy. We have limited the search, to target our audience as good the literature allowed us. Clark et al. clearly states that the target audience should be as specific as possible [9, 10]. The articles represented in this review were selected with focus on postgraduate learners; further analysis will be required to determine the applicability of our Postgraduate ME Model to other audiences. Even if e-learning developers reject our model, we feel that it is better to have reasons for not using a model than to have no model at all [71].

## Conclusion

In summary, our Postgraduate ME Model aimed to provide a practical framework that can be used to build postgraduate medical e-learning programs that are learner centred, interactive, well planned and designed, based on cognitive load theory, and easy to maintain. E-learning should be about the learner, not the technology. Our proposed model may guide e-learning designers who are developing quality e-learning targeted to postgraduates in medicine. Our six-domain model is unique in that it combines the technical requirements from industry standards with the critical aspects of content and interaction from learning theories.

The next step is for research to validate these domains with international experts to determine if they are beneficial to postgraduate real-world e-learning. It would be interesting to know if postgraduate e-learning experts agree with our description of qualitative e-learning, based on their experience. Another important question is whether a model such as this could actually be used in practice when developing e-learning platforms. Ultimately, we would like to know whether e-learning based on our Postgraduate ME Model will reproducibly improve learner motivation.

## Abbreviations

CLT, Cognitive load theory; ISO, International standardization organization; Postgraduate ME Model, Postgraduate Medical Education Model
